# Trends in low global warming potential inhaler prescribing: A UK-wide cohort comparison from 2018–2024

**DOI:** 10.1038/s41533-025-00415-z

**Published:** 2025-02-20

**Authors:** Simon M. Barry, Gareth R. Davies, Julian Forton, Sarah Williams, Richard Thomas, Paul Paxton, Grace Moore, Chris R. Davies

**Affiliations:** 1 Respiratory Medicine, Cardiff and Vale UHB, Cardiff, UK; 2 School of Medicine, Cardiff University, Cardiff, UK; 3 Institute of Clinical Science and Technology, Cardiff, UK; 4 Children’s Hospital for Wales, Cardiff, UK; 5 Value-in-Health Wales Sustainable Primary Care Lead, Cardiff, UK; 6 Co-Founder Greener Practice Wales, Abergavenny, UK; 7 Public Health Scotland, Edinburgh, UK

**Keywords:** Health policy, Therapeutics

## Abstract

We performed a retrospective cohort analysis comparing trends in low global warming potential (GWP) inhaler prescribing in primary care in England, Scotland, Wales and Northern Ireland between 2018 and 2024 using national prescribing data. There was little change in England, a reduction from 36.6–31.0% in Scotland, a reduction from 36.7–33.2% in Northern Ireland, and an increase from 30.8–41.1% in Wales. Only in Wales was there a simultaneous reduction in high GWP inhalers and an increase in low GWP inhalers. Over the time period of the study there has been a saving of 20,303 tonnes of carbon dioxide equivalent in Wales.

Current propellants from metered dose inhalers (MDI) are hydrofluorocarbons (HFA–134a and HFA–227ea) that are powerful global warming gases contributing approximately 3.1% of the total carbon footprint on the NHS^1^. By contrast, low global warming potential (GWP) inhalers comprising dry powder inhalers (DPI) and soft mist inhalers (SMI) have much lower carbon footprints. The United Kingdom lags far behind the rest of Europe with the proportion of low GWP inhalers being only 30%, versus a European average of 50%, with exemplar countries such as Sweden attaining 87%^2^. As a consequence, national bodies in the UK have promoted the preferential prescription of low GWP inhalers^3^. A move to more environmentally sustainable inhalers is also strongly supported by patients with airways disease^4^. A 2018 UK government report set a target that 50% of inhalers would be low GWP by 2022^5^, but by that time there had been no real change in any country. Wales now has a goal that 80% of inhalers will be low GWP by 2025^6^, in Scotland the target is a 70% reduction in the carbon footprint from inhalers by 2028^7^, and in England a 50% reduction by 2030^8^. There is no specific target for Northern Ireland.

Factors promoting preferential low GWP inhaler prescribing include national guidelines, incentivised quality improvement projects, national prescribing indicators and the influence of greener practice organisations. In Wales, an integrated respiratory toolkit comprising national asthma and chronic obstructive pulmonary disease (COPD) guidelines with icons denoting the carbon footprint of inhalers^9^, educational and quality improvement modules for healthcare professionals, and applications (apps) for asthma and COPD patients were created by the Institute of Clinical Science and Technology (ICST) in collaboration with the Respiratory Health Implementation Group, NHS Wales and implemented across Wales utilising a digital implementation framework^10^ starting in the autumn of 2021. The Welsh paediatric asthma guidelines suggest switching to dry powder inhalers at age six for those that can use the devices.

We compare the inhaler switching trends between the four nations over the study period and consider the factors promoting this process.

## Methods

Primary care data for low and high GWP inhalers prescribed within the periods under consideration were collected monthly in all four nations using a standard basket of low GWP inhalers. A source of inhaler type e.g. DPI, MDI, SMI and carbon footprint values for individual inhalers was identified from PrescQIPP^11^. Data was collected in Wales from CASPA (Comparative Analysis System for Prescribing Audit) by All Wales Therapeutics and Toxicology Centre (AWTTC), in England from ePACT2 by AWTTC, in Scotland from the prescribing information system (PIS) by Paul Paxton and from Northern Ireland by the Family Practitioner Services (FPS) Directorate of the Business Services Organisation. Data on the carbon footprint in Wales from inhaler prescribing was calculated by then multiplying the assigned carbon footprint of an inhaler by the number of items within the dataset. Where a short acting B2 agonist (SABA) was prescribed generically, it was assumed that it was high GWP salbutamol.

The primary outcome was the change in the percentage of low GWP inhalers by country from 2018–2024.To identify any statistically significant changes in the linear trends of percent low GWP inhalers we used Joinpoint regression analysis software^12^. Joinpoint identifies the best fitting model from the range of joinpoint models evaluated. All of the models assumed constant variance with first order autocorrelation estimated from the data and the maximum number of possible joinpoints constrained to 3. All other settings were as per the system defaults with the significance level of 0.05. The data were exported to Stata^13^ for production of the plots. Joinpoint regression models were also used to identify the best fitting regression lines for the actual number of prescriptions of each inhaler type. The average daily prescription counts were based on working days and therefore took account of weekends and bank holidays.

## Results

**Fig. 1:**
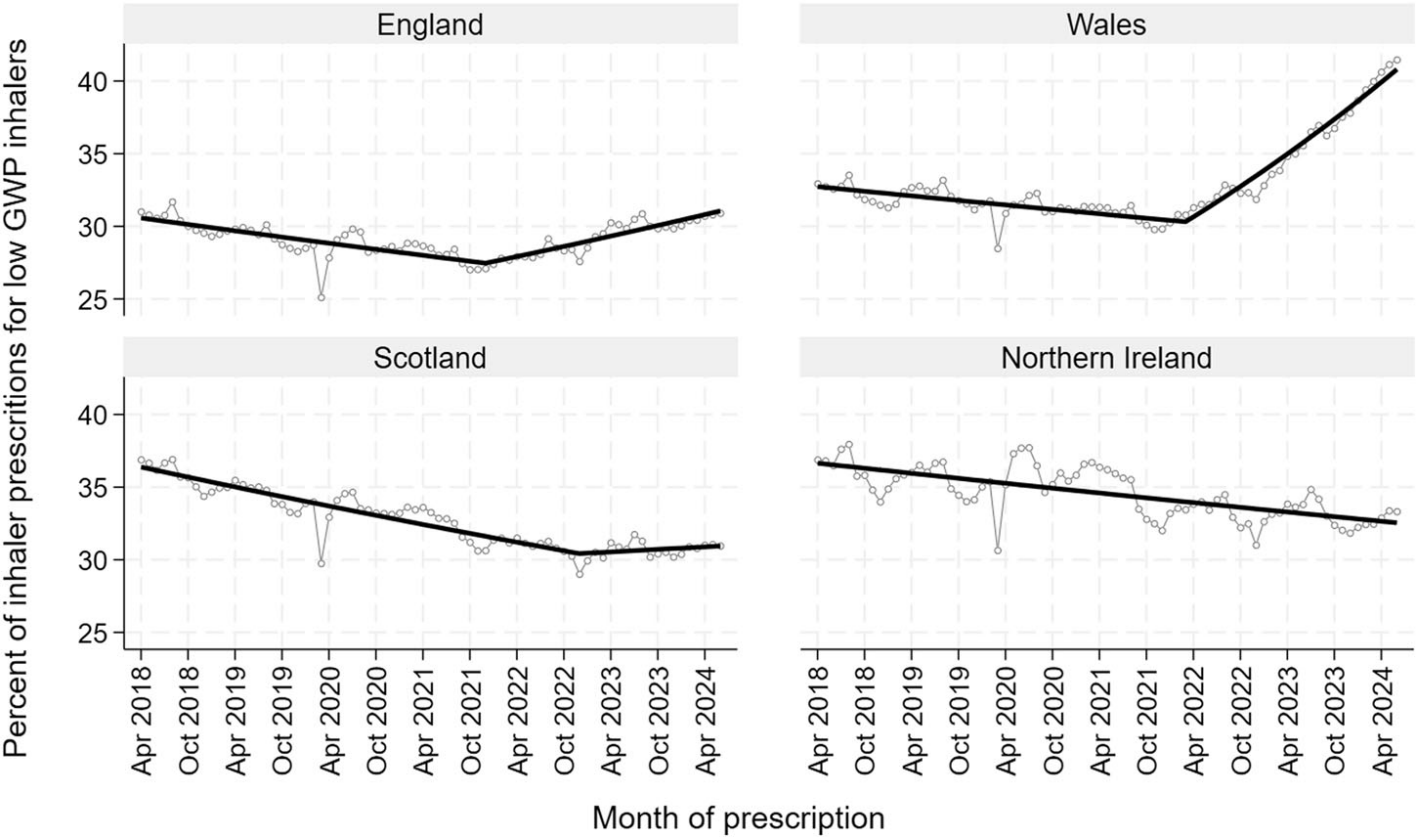
The percentage of all primary care inhalers that were low GWP in England, Scotland, Wales and Northern Ireland between April 2018 and January 2024.

The best fitting Joinpoint models for three nations had two distinct trends, with changes occurring in December 2021 for England, March 2022 for Wales, and December 2022 for Scotland (Fig. 1). England, Wales and Scotland ended on an upward trend, but only Wales had a higher percentage of low GWP inhalers at the end of the study period than at the start of it.

The rates of change were significant for all trends for all nations except Scotland after December 2022 with the greatest rate of change being in Wales from March 2022 (Fig. 1 & Supplementary Table 1.1)

**Fig. 2:**
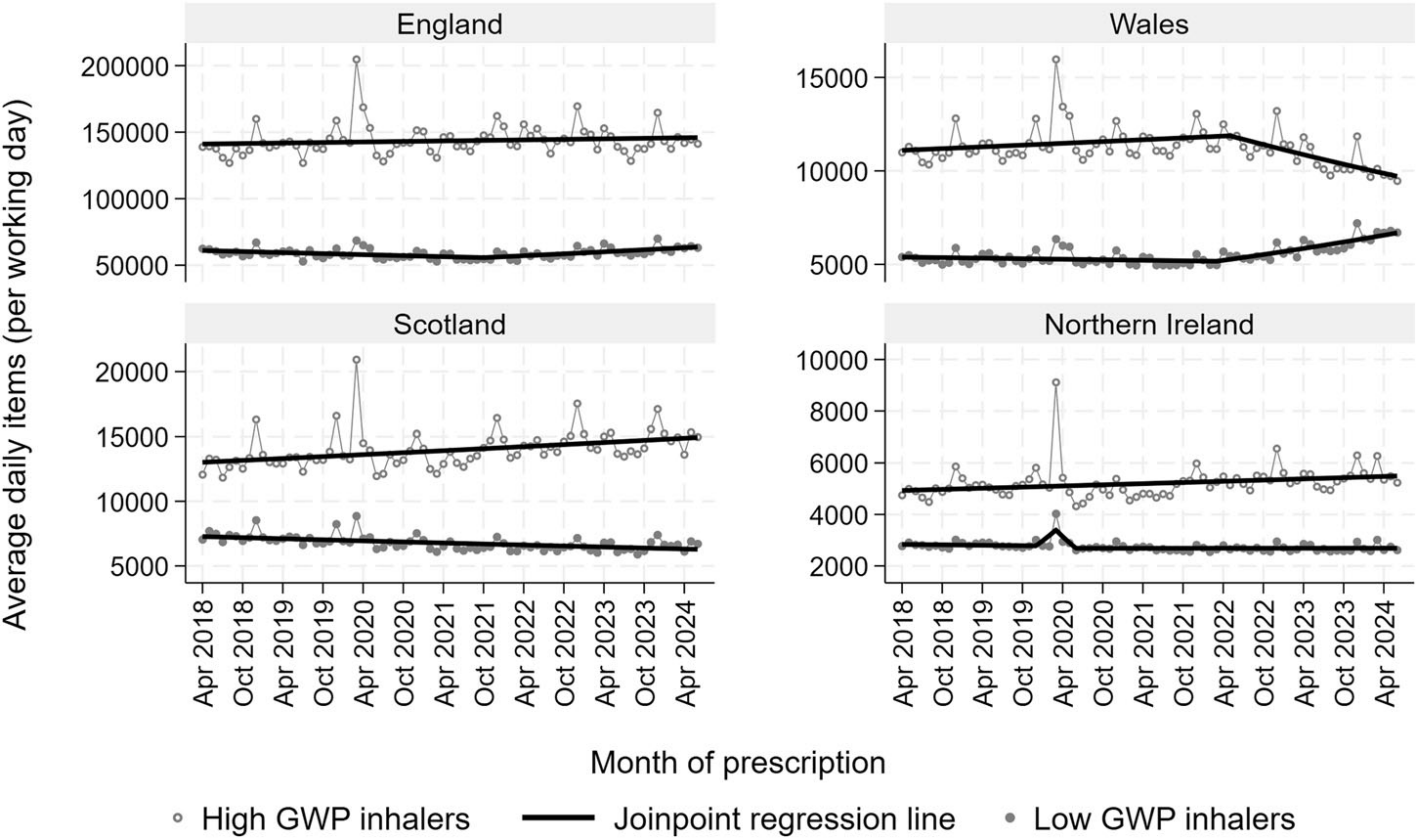
Trends for low and high GWP inhalers in England, Scotland, Wales and Northern Ireland between April 2018 and January 2024.

When the trends for the average prescriptions for high and low GWP inhalers were compared between the countries (Fig. 2 & Supplementary Tables 2.1, 2.2), only in Wales was there a simultaneous reduction in high GWP inhalers and increase in low GWP inhalers. In March 2020 there was a notable, but temporary increase in high GWP inhalers corresponding to the onset of the COVID pandemic in all four countries. In Wales, this was accounted for predominantly by a large increase in the prescription of short acting B_2_ agonist (SABA) MDIs, with much smaller increases in inhaled corticosteroid (ICS) MDI, ICS DPI and inhaled corticosteroid/long acting B_2_ agonist (ICS/LABA) DPI (AWTTC data).

When comparing prescribing data from September 2018 to August 2019 with September 2023 to August 2024 in Wales, there was a reduction in the carbon footprint from inhalers of 20,303 tonnes of CO_2_ equivalent.

The proportions of low GWP inhalers were reducing in England, Wales and Scotland until various points in 2021/22 when the trends reversed. The proportion of low GWP inhalers in Northern Ireland declined across the whole period under study. It is notable that a national incentivised quality improvement project to switch inhalers to low GWP devices ran in England from October 2021 until January 2022 and again from April 2022 until March 2023, and in Wales from October 2022 until March 2024, but did not exist in Scotland or Northern Ireland. In addition, greener practice groups launched an open access website in April 2022^14^ that highlighted the green agenda in inhaler prescribing amongst other environmental issues. However, the change in Wales started before these two interventions and has been greater than in England. Wales implemented a national respiratory toolkit across the whole of primary care promoting the green agenda. This toolkit included updated guidelines denoting inhaler carbon footprint delivered in digital and print format to every practice in Wales from the autumn of 2021. In addition, a coordinated campaign was initiated in the same period including webinars and video-based updates delivered to 13,000 healthcare professionals (HCP) on the digital platform and 18,200 respiratory app users with the aim of motivating both patients and HCP to switch to low GWP inhalers (Supplementary Table 3). The lack of change in England from either the greener practice site or the incentivised QI project and the timing of implementation of the respiratory toolkit in Wales suggests that it was the latter that was the most important factor in creating behaviour change. The limited effect of the incentivised QI project is unsurprising since the evidence that financial incentives improve outcomes is weak^15,16^. We have noted a significant reduction in the carbon footprint from our inhaler switching of 20,303 tonnes in Wales. Whilst this is a small step in contributing to combatting the climate emergency, the wider societal cost of carbon has recently been calculated to $185 (or £145) per tonne of carbon dioxide^17^. By this analysis, we have saved £2.9 million by our actions in Wales.

There are a number of limitations. We have reported only on primary care prescribing data, whilst being aware that this represents the vast majority of all inhaler prescribing. We cannot be sure of the relative contributions of different influences in creating change, although we note the lack of behaviour change in the other UK countries where various factors were at play. We have not measured changes to moderate GWP short acting B2 agonist inhalers (Salamol from salbutamol/Ventolin) which may have been important in reducing the carbon footprint of inhalers in all countries. We also recognise that future generations of propellants for MDI will have lower GWP which will change the dynamic of inhaler prescribing. It is clear that additional factors to national guideline policies such as the toolkit approach adopted in Wales are necessary to create widespread behaviour change in inhaler prescribing.

## Supplementary information

Supplementary Table 3

Supplementary information
