# Supplementary material for: Trends in low global warming potential inhaler prescribing: A UK-wide cohort comparison from 2018–2024
Source: NPJ Prim Care Respir Med. 2025 Feb 20;35:9. doi: 10.1038/s41533-025-00415-z (PMC11842734; doi:10.1038/s41533-025-00415-z)
Supplement: Supplementary file 1 — Supplementary Table 3 [file 41533_2025_415_MOESM1_ESM.docx]

Supplementary Table 3

Engagement with the NHS Wales Sustainable Prescribing Programme

To ensure the messaging provided to healthcare professionals and patients is optimised, campaigns are methodically managed as part of a system that uses behaviour change methodologies to ensure users have the capability, opportunity and motivation to make the switch to more sustainable inhalers. Access to information on the toolkits is restricted to users with accounts, allowing for specific, targeted communication to known users who will benefit, and the ability to have data returned, which is an outcome of creating a trusted two-way relationship. Consequently, the reported numbers of touchpoints, carry significantly higher value, that compounds over time- rather than a simple one-way transaction.

|  | Data |
| --- | --- |
| Campaigns | **September 2021**  **Guideline adaptations and Programme publishing**  In September 2021, guidelines were adjusted to promote sustainable prescribing practices, accompanied by an educational program focused on informing healthcare professionals about the rationale for medication switches, giving them the motivation to switch, and providing them with the necessary skills and opportunities to help their patients make the transition.  This programme was published to the All Wales platform, which has 13,000 healthcare professionals in 98% of GP Practices across Wales, including those in secondary care, primary care, pharmacy, and community respiratory teams, all who hold direct authority over inhaler prescriptions for patients.    **December 2021**  **Sustainable Prescribing Launch**  During a three-week period, 32,000 priming emails were distributed to 8,000 healthcare professionals spanning 98% of GP practices in Wales. This campaign provided healthcare professionals with the necessary background and motivation for the required switch, along with opportunities to enhance their skills in assisting patients with the transition.  Furthermore, within the same timeframe, 13,000 emails were sent to 4,000 patients across 99% of GP practices throughout Wales, who use the inhalers subject to the transition. This campaign used behaviour change methodologies to provide patients with the motivation, opportunity, and capability necessary to transition to environmentally-friendly inhalers.  The average open rate for these emails was: 41%  Compared to the industry average of 22%  As part of the initiative, healthcare professional and patient events were organised and promoted to all healthcare professionals and app users throughout Wales.  Recordings of the events are available on the platform, allowing individuals to view them at their convenience. This means an increased number of healthcare professionals can engage with the campaign, benefiting the 14,000 current users of the platform.  **January 2022**  **Guideline send out**  A total of 4,000 emails were dispatched to 2,000 healthcare professionals who prescribe inhalers to patients across 98% of GP Practices in Wales, notifying them about the campaign, and the forthcoming delivery of physical copies of sustainable prescribing guidelines to their respective practices.  The physical guidelines were distributed as supplements to the sustainable prescribing program, aiming to equip healthcare professionals with the capability and opportunity to assist their patients in making the transition during consultations. These guidelines included QR codes that linked to educational programs providing context about the switch, enhancing healthcare professionals' ability to support their patients effectively.  15,450 physical copies of the sustainable prescribing guidelines were included in packages sent to all GP Surgeries across Wales.  Subsequent follow-up emails were sent to the same 2,000 healthcare professionals to confirm receipt of the updated guidelines and to accommodate any additional requests.  ICST distributed an extra 3,417 guidelines to those requesting more copies.  **March 2023**  **QI project launch**  The Quality Improvement (QI) project was initiated in March 2023 to provide healthcare professionals with the necessary resources and tools to facilitate the transition of patients attending reviews in their practice. This initiative aimed to enhance healthcare professionals' capability, thereby increasing the likelihood of altering their prescribing behaviours towards more sustainable practices.  During a three-week period, 6,000 priming emails were dispatched to 2,000 primary care-based healthcare professionals, covering 98% of GP practices in Wales. These professionals possess the direct authority to prescribe inhalers to patients and can facilitate the transition to environmentally friendly inhalers.  Open rate: 35.2%  As part of the launch an event was held for Healthcare Professionals in primary care, and this was promoted to 2,000 primary care based healthcare professionals in Wales.  Recordings of the events are available on the platform, allowing individuals to view them at their convenience.  **March 2023**  **Getting the most from your inhalers**  This patient campaign focused on educating individuals about their inhalers and their potential environmental impact. It also targeted the thousands of new users who had signed up within the 15 months following the campaign's launch. The initiative aimed to provide users with the skills to discuss environmental concerns with their healthcare professionals, referencing a trusted information source. Additionally, it offered users the opportunity to address any questions they might have regarding their inhalers.  During a three-week period, 36,200 emails were sent to 11,000 patients across 99% of GP Practices across Wales who utilise the inhalers undergoing the switch, prompting them to visit their GP and review their prescribed inhalers.  Open rate: 53.5%  As part of this campaign, three events were organised for the 11,000 app users, connecting them with trusted experts in respiratory disease. These events aimed to encourage patients to make the switch to greener inhalers and addressed commonly asked questions regarding the transition.  Recordings of the events are available on the Healthhub website and are linked as a resource from the apps, allowing individuals to view them at their convenience. This accessibility leads to an increase in the number of patients engaging with the campaign, benefiting the 18,200 current users of the apps.  **April 2023**  **Paediatric Asthma Guideline Launch**  The aim of this campaign was to educate healthcare professionals about the updated guidelines in pediatric asthma treatment and provide them with the necessary tools and resources to transition their patients to environmentally friendly inhalers.  During a three-week period, 53,000 emails were dispatched to 11,000 healthcare professionals in Wales, covering 98% of GP practices in Wales. These professionals possess the direct authority to prescribe inhalers to patients and can facilitate the transition to environmentally friendly inhalers.  Open rate: 26.7%  As part of the campaign, an event was held for Healthcare Professionals in primary care, and this was promoted to 11,000 healthcare professionals in Wales.  Recordings of the events are now available on the platform, allowing individuals to view them at their convenience. This accessibility leads to an increase in the number of healthcare professionals engaging with the campaign, benefiting the **13,000 current users** of the platform.  **January 2024**  **New COPD Guideline Launch**  The aim of this campaign was to educate healthcare professionals about the updated guidelines in COPD treatment and provide them with the necessary tools and resources to transition their patients to environmentally friendly inhalers.  During a three-week period, 25,400 emails were dispatched to 5000 healthcare professionals in Wales, covering 98% of GP practices in Wales. These professionals possess the direct authority to prescribe inhalers to patients and can facilitate the transition to environmentally friendly inhalers.  Open rate: **32%**  As part of the campaign, an event was held for Healthcare Professionals in primary care, and this was promoted to 5,000 healthcare professionals in Wales.  Recordings of the events are now available on the platform, allowing individuals to view them at their convenience. This accessibility leads to an increase in the number of healthcare professionals engaging with the campaign, benefiting the **13,000 current users** of the platform. |
| Background activity | Since December 2021, the All Wales Platform has prominently displayed promotional banners for its sustainable prescribing campaign on its homepage, featuring a link to the program and QI project. This banner aimed to raise awareness of the campaign among users and provide them with easy access to further information, as well as the necessary skills and resources. With 50,000 logins recorded during this period, it can be inferred that the banner was viewed at least this number of times, ensuring widespread visibility of the campaign's objectives and resources.  In January 2022, a total of 18,867 physical copies of the sustainable prescribing guidelines were distributed to all GP Practices in Wales, ensuring coverage of 100% of practices. These guidelines were disseminated in physical form to closely align with standard practice and were prominently displayed in clinical rooms across the nation. General practitioners routinely consult these guidelines when prescribing medications for both new and existing patients, thereby integrating sustainable prescribing principles into their everyday clinical decision-making processes.  Patients using the apps receive consistent access to essential knowledge, resources, and skills to prioritise inhaler prescribing. They are regularly prompted to schedule reviews with their healthcare professionals, which involves assessments of their prescribed inhalers and potential transitions to greener alternatives.  Moreover, since its launch in December 2021, the education page on the patient apps, featuring the "how eco-friendly are your inhalers?" video, has had over 10,000 views. This educational content offers users context and motivation regarding the switch to more sustainable inhalers. It addresses frequently asked questions and aids users in developing the skills necessary to engage their healthcare professionals. |
