# Supplementary material for: Trends in low global warming potential inhaler prescribing: A UK-wide cohort comparison from 2018–2024
Source: NPJ Prim Care Respir Med. 2025 Feb 20;35:9. doi: 10.1038/s41533-025-00415-z (PMC11842734; doi:10.1038/s41533-025-00415-z)
Supplement: Supplementary file 2 — Supplementary information [file 41533_2025_415_MOESM2_ESM.docx]

**Supplementary information for Joinpoint analysis**

**Figure 1**

**Supplementary Table 1.1: Joinpoint analysis, percent of inhaler prescriptions for low GWP inhalers**

|  | Low GWP inhalers  (period %) | | | Joinpoint analyses (Apr18 – Jun24) | | | | | | | | | | | | | |
| --- | --- | --- | --- | --- | --- | --- | --- | --- | --- | --- | --- | --- | --- | --- | --- | --- | --- |
|  |  |  |  | Trend 1 | | | |  | Trend 2 | | | |  | Overall | | | |
| Country | Apr18 to Jun18 | Apr24 to Jun24 | change | Period | MPC | 95% CI | p |  | Period | MPC | 95% CI | p |  | Period | AMPC | 95% CI | p |
| England | 31.01 | 30.81 | -0.20 | Apr18 -  Dec21 | -0.24 | -0.42 to -0.13 | <0.01 |  | Dec21 -  Jun24 | 0.41 | 0.23 to 0.86 | <0.01 |  | Apr18 -  Jun24 | 0.02 | -0.04 to 0.08 | 0.42 |
| Wales | 30.81 | 41.07 | 10.26 | Apr18 -  Mar22 | -0.16 | -0.25 to -0.09 | <0.01 |  | Mar22 -  Jun24 | 1.11 | 0.93 to 1.35 | <0.01 |  | Apr18 -  Jun24 | 0.30 | 0.25 to 0.34 | <0.01 |
| Scotland | 36.57 | 31.02 | -5.55 | Apr18 -  Dec22 | -0.32 | -0.57 to -0.23 | 0.04 |  | Dec22 -  Jan24 | 0.09 | -0.27 to 2.44 | 0.47 |  | Apr18 -  Jan24 | -0.22 | -0.31 to -0.15 | <0.01 |
| Northern Ireland | 36.73 | 33.20 | -3.53 | Apr18 -  Jun24 | -0.16 | -0.23 to -0.09 | <0.01 |  | - | - | - | - |  | Apr18 -  Jun24 | -0.16 | -0.23 to -0.09 | <0.01 |

**NOTES**

The MPC (monthly percentage change) and AMPC (average monthly percentage change) are calculated by the JoinPoint software. They are both estimates of the rate of linear change in the observed trends. The MPC is the rate of linear change within each separate JoinPoint segment. The AMPC is the rate of linear change across all the data points.

Therefore, a positive MPC or AMPC indicates an increase over time, a negative one indicates a decrease. The confidence intervals and p value indicate whether the increase/decrease is truly different from zero.

The MPC and AMPC are relative rather than absolute measures of the rate of change. As an illustration, in Wales between March 2022 and June 2024 the MPC of 1.11 indicates that the percentage of low GWP inhalers for any one month in that period would be 1.00 + (1.11/100) = 1.0111 times the percentage of the preceding month.

**Figure 2**

**Supplementary Table 2.1: Joinpoint analysis, low GWP inhalers – trends in numbers**

|  | Low GWP inhalers  (daily average) | | | Joinpoint analyses (Apr18 – Jun24) | | | | | | | | | | | | | |
| --- | --- | --- | --- | --- | --- | --- | --- | --- | --- | --- | --- | --- | --- | --- | --- | --- | --- |
|  |  |  |  | Trend 1 | | | |  | Trend 2 | | | |  | Trend 3 | | | |
| Country | Apr18 to Jun18 | Apr24 to Jun24 | % change | Period | MPC | 95% CI | p |  | Period | MPC | 95% CI | p |  | Period | MPC | 95% CI | p |
| England | 61,709 | 63,532 | 2.95 | Apr18 -  Oct21 | -0.22 | -0.45 to -0.08 | <0.01 |  | Oct21 -  Jun24 | 0.42 | 0.22 to 0.82 | <0.01 |  | - | - | - | - |
| Wales | 5,419 | 6,739 | 24.36 | Apr18 -  Mar22 | -0.09 | -0.27 to 0.04 | 0.18 |  | Mar22 -  Jun24 | 0.96 | 0.67 to 1.45 | <0.01 |  | - | - | - | - |
| Scotland | 7,419 | 6,573 | -11.40 | Apr18 -  Jun24 | -0.20 | -0.27 to -0.12 | <0.01 |  | - | - | - | - |  | - | - | - | - |
| Northern Ireland | 2,838 | 2,665 | -6.10 | Apr18 -  Dec19 | -0.10 | -0.46 to 0.15 | 0.37 |  | Dec19 - Mar20 | 6.89 | 2.70 to 9.24 | <0.01 |  | Mar20 -  Jun20 | -7.54 | -9.28 to -3.52 | <0.01 |

|  | Joinpoint analyses (Apr18 – Jun24) | | | |
| --- | --- | --- | --- | --- |
|  | Trend 4 | | | |
| Country | Period | MPC | 95% CI | p |
| England | - | - | - | - |
| Wales | - | - | - | - |
| Scotland | - | - | - | - |
| Northern Ireland | Jun20 -  Jun24 | 0.00 | -0.07 to 0.09 | 0.84 |

**Supplementary Table 2.2: Joinpoint analysis, high GWP inhalers – trends in numbers**

|  | High GWP inhalers  (daily average) | | | Joinpoint analyses (Apr18 – Jun24) | | | | | | | | |
| --- | --- | --- | --- | --- | --- | --- | --- | --- | --- | --- | --- | --- |
|  |  |  |  | Trend 1 | | | |  | Trend 2 | | | |
| Country | Apr18 to Jun18 | Apr24 to Jun24 | % change | Period | MPC | 95% CI | p |  | Period | MPC | 95% CI | p |
| England | 138,676 | 142,663 | 2.88 | Apr18 -  Jun24 | 0.05 | -0.04 to 0.13 | 0.32 |  | - | - | - | - |
| Wales | 11,128 | 9,668 | -13.11 | Apr18 -  May22 | 0.14 | -0.03 to 0.51 | 0.10 |  | May22 -  Jun24 | -0.80 | -2.26 to -0.33 | <0.01 |
| Scotland | 12,869 | 14,617 | 13.58 | Apr18 -  Jun24 | 0.19 | 0.08 to 0.29 | <0.01 |  | - | - | - | - |
| Northern Ireland | 4,889 | 5,362 | 9.67 | Apr18 -  Jun24 | 0.15 | 0.04 to 0.26 | 0.01 |  | - | - | - | - |
